# Association Between Metabolic Syndrome and Prostate Cancer: A Systematic Review

**DOI:** 10.3390/cancers18121955

**Published:** 2026-06-16

**Authors:** Supriya Peshin, Ehab Takrori, Bhavesh Mohan Lal, Sakshi Singal, Konstantinos Arnaoutakis, Anuradha Kunthur, Shi Ming Tu

**Affiliations:** 1 Department of Internal Medicine, Norton Community Hospital, Ballad Health System, Norton, VA 24273, USA; 2 College of Medicine, Alfaisal University, Riyadh 11533, Saudi Arabia; etakrori@gmail.com; 3 Department of Internal Medicine, University of Arkansas for Medical Sciences, Little Rock, AR 72205, USA; bmohanlal@uams.edu (B.M.L.); shiming.tu@renown.org (S.M.T.); 4 Department of Hematology & Oncology, East Tennessee State University, Johnson City, TN 37614, USA; singal@mail.etsu.edu; 5 Division of Hematology and Medical Oncology, University of Arkansas for Medical Sciences, Little Rock, AR 72205, USA; karnaoutakis@uams.edu

**Keywords:** metabolic syndrome, prostate cancer, insulin resistance, cardiometabolic risk

## Abstract

*Background/Objectives*: Metabolic syndrome is a prevalent global health concern characterized by central obesity, insulin resistance, hypertension, and dyslipidemia. Growing evidence suggests that metabolic dysregulation may influence prostate cancer risk and disease behavior; however, findings across studies remain inconsistent. This systematic review aimed to evaluate the association between metabolic syndrome and prostate cancer risk, clinicopathologic characteristics, and disease outcomes. *Methods*: A systematic literature search was conducted in PubMed/MEDLINE from database inception through the final search update on 10 January 2026, with supplementary searches of Google Scholar, Wiley Online Library, the *Clinical Oncology* journal collection, and reference lists. Observational studies assessing the relationship between metabolic syndrome and prostate cancer outcomes were eligible for inclusion. Two reviewers independently screened titles and abstracts, followed by full text review to determine eligibility. Discrepancies were resolved through consensus. Risk of bias was assessed using design-specific tools, including the Newcastle–Ottawa Scale, Cochrane Risk of Bias 2 tool, and Joanna Briggs Institute checklist. Findings were synthesized narratively because of heterogeneity in study design, metabolic syndrome definitions, populations, outcomes, and effect estimates. *Results*: A total of 1304 records were identified across all databases. After duplicate assessment, records underwent title and abstract screening, followed by full text assessment. Twenty-four studies, ranging from small clinic-based cohorts to large population-based cohorts exceeding 5 million participants, met the inclusion criteria and were included in the qualitative synthesis. Most studies were cohort or case–control designs conducted in North America, Europe, and Asia. Overall, evidence for a uniform association between composite metabolic syndrome and prostate cancer incidence was inconsistent. Associations appeared more suggestive for clinically significant, high-grade, or adverse pathological features, although findings varied by study design, metabolic syndrome definition, detection context, and adjustment level. Several studies suggested that individual components of metabolic syndrome, particularly obesity and insulin resistance, may be key drivers of these associations. *Conclusions*: Available evidence suggests a heterogeneous relationship between metabolic syndrome, related metabolic abnormalities, and prostate cancer outcomes. Evidence for a uniform association with overall prostate cancer incidence was inconsistent, whereas signals were more frequently reported for clinically significant, high-grade, or adverse pathological features. However, these findings should be interpreted cautiously because of observational study designs, heterogeneous MetS definitions, detection context, and variable adjustment. Further well-designed prospective studies are needed to clarify temporality, causal pathways, and the contributions of individual metabolic components.

## Simple Summary

Metabolic syndrome is a group of conditions that includes obesity, high blood pressure, abnormal cholesterol levels, and impaired blood sugar control. These conditions may influence prostate cancer development and behavior, but published studies have reported inconsistent findings. This systematic review summarizes available evidence on whether metabolic syndrome is associated with prostate cancer risk, aggressive tumor features, recurrence, and survival. Overall, the evidence suggests that metabolic syndrome may be more strongly linked to clinically significant or aggressive prostate cancer than to prostate cancer incidence alone. The relationship appears to vary by population, study design, and how metabolic syndrome is defined, with obesity and insulin resistance likely playing important roles. More standardized prospective studies are needed to clarify whether improving metabolic health can reduce prostate cancer risk or improve outcomes.

## 1. Introduction

Prostate cancer is one of the most frequently diagnosed malignancies in men worldwide and remains a major contributor to cancer-related morbidity and mortality. Global estimates from the International Agency for Research on Cancer (IARC) estimate there were approximately 1.47 million new prostate cancer cases and 397,430 deaths globally in 2022, underscoring a substantial public health burden with marked geographic variation in incidence and mortality [1,2]. Identifying modifiable risk factors that may influence prostate cancer development and aggressiveness remain a priority.

Metabolic syndrome (MetS) is a prevalent cardiometabolic disorder characterized by a constellation of interrelated abnormalities, typically including central obesity, dyslipidemia (elevated triglycerides and/or low HDL cholesterol), elevated blood pressure, and impaired glucose regulation and insulin resistance [3,4]. Despite broad agreement regarding its components, MetS definitions differ across major criteria frameworks (e.g., NCEP ATP III and IDF), resulting in variability in case classification across studies and populations [3,5]. This definitional heterogeneity is especially relevant when evaluating cancer outcomes, where differences in thresholds for waist circumference, glucose, and lipid parameters may alter observed associations.

A biologically plausible link between metabolic dysfunction and prostate carcinogenesis has been proposed. Hyperinsulinemia and insulin resistance may promote tumor initiation and growth through the insulin-IGF axis, which is implicated in prostate cell proliferation and survival signaling [6,7]. In parallel, obesity-related chronic low-grade inflammation can contribute to a pro-tumorigenic microenvironment via cytokine and immune pathway dysregulation [8]. Adipose-derived mediators (adipokines) have also been proposed as potential intermediates connecting adiposity and metabolic disturbance to prostate cancer risk and progression [9].

Interpretation of epidemiologic findings is further complicated by detection-related factors. Multiple studies have demonstrated that obesity is associated with lower measured PSA concentrations, in part attributable to plasma volume-related hemodilution, which may reduce screening sensitivity and delay diagnosis [10,11]. As a result, metabolic dysfunction could appear variably associated with incidence versus advanced stage or aggressive disease depending on screening practices, baseline PSA distributions, and healthcare access.

Observational evidence evaluating MetS and prostate cancer outcomes has remained inconsistent. Some contemporary prospective data from large population cohorts (including UK Biobank analyses) suggest associations between MetS and prostate cancer risk [12]. Other studies report stronger links with aggressive disease phenotypes across racial groups [13]. In biopsy-based settings, MetS has also been associated with higher risk of prostate cancer detection following certain premalignant histopathologic findings [14]. However, null associations are reported in other cohorts, particularly for recurrence and survival endpoints, and the extent to which individual MetS components drive risk versus the clustered syndrome itself remains uncertain.

Given these inconsistencies and the substantial heterogeneity in MetS definitions, populations, screening contexts, and endpoints, a systematic synthesis of the evidence is warranted. This systematic review aims to evaluate the association between metabolic syndrome and prostate cancer risk, clinicopathologic characteristics (grade/stage), and disease outcomes, while highlighting sources of heterogeneity and identifying gaps to guide future prospective and mechanistic research.

## 2. Methodology

### 2.1. Protocol and Registration

This systematic review was prospectively registered in PROSPERO (Registration No. CRD420261278024). The methods were pre-specified in the protocol, and no amendments were made.

### 2.2. Reporting Guidelines

**Figure 1:**
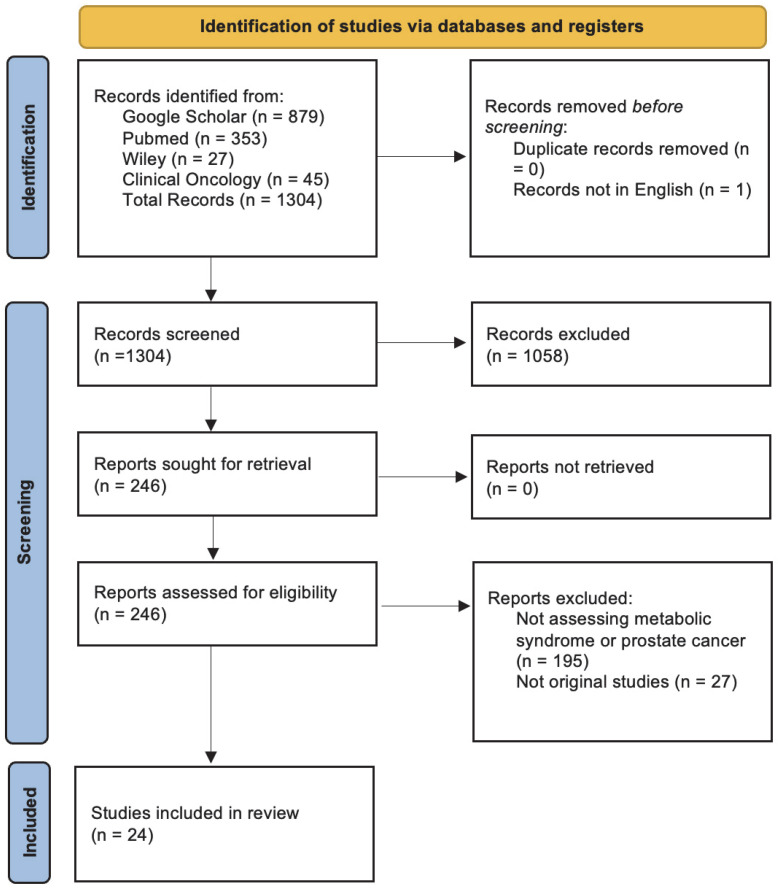
PRISMA Flow Diagram.

This systematic review was conducted and reported in accordance with the Preferred Reporting Items for Systematic Reviews and Meta-Analyses (PRISMA) 2020 statement [15]. A PRISMA flow diagram was used to document study selection (Figure 1), including records identified from each source, duplicate assessment, title/abstract screening, full-text assessment, exclusion reasons, and final included studies. The completed PRISMA 2020 checklist is provided in the Supplementary Materials.

### 2.3. Eligibility Criteria

To ensure data relevance, a research question was developed using the PICO-TT framework, focusing on adult men with or without metabolic syndrome and evaluating its association with prostate cancer risk, clinicopathologic characteristics (e.g., grade and stage), and disease outcomes (e.g., recurrence and survival). The review included prospective and retrospective cohort studies, case–control studies, cross-sectional studies, randomized controlled trials, and multicenter observational studies that assessed metabolic syndrome (or clearly defined metabolic syndrome components meeting a stated diagnostic framework) in relation to prostate cancer outcomes. Studies were excluded if they were reviews, editorials, letters, case reports/series, conference abstracts without sufficient extractable data, animal or in vitro studies, or if they did not report relevant prostate cancer endpoints or provided insufficient data to determine the association. The inclusion and exclusion criteria are summarized in Table 1.

### 2.4. Information Sources

A systematic literature search was conducted using PubMed/MEDLINE as the primary biomedical database, with supplementary searches of Google Scholar, Wiley Online Library, the Clinical Oncology journal collection, and reference lists of included studies, from database inception through the final search update on 10 January 2026. To ensure completeness, the reference lists of all included articles and other relevant papers identified during screening were also manually reviewed to capture any additional eligible studies not retrieved through the database searches.

PubMed/MEDLINE was selected as the primary biomedical bibliographic database because it provides broad coverage of epidemiologic and clinical research relevant to metabolic syndrome and prostate cancer. Wiley Online Library was searched as a supplementary source to capture relevant full-text articles hosted on the Wiley platform that may not be consistently retrieved through PubMed indexing alone. In addition, we searched *Clinical Oncology* to ensure coverage of prostate cancer focused literature. To further reduce the risk of missed studies, we also performed supplementary searches using Google Scholar and screened reference lists of included articles.

### 2.5. Search Strategy

A systematic search strategy was developed to identify studies evaluating the association between metabolic syndrome and prostate cancer outcomes. Search terms were constructed using combinations of key concepts related to the exposure (metabolic syndrome) and the outcome (prostate cancer and related clinical endpoints) and were adapted to the syntax and functionality of each database. For PubMed, both MeSH terms and free-text keywords were used, whereas Wiley Online Library, Google Scholar, and Clinical Oncology were searched using free-text keywords. The complete keywords, search strings, filters, and the number of records retrieved from each database are summarized in Table 2.

The complete PubMed/MEDLINE search strategy, including MeSH terms, Boolean operators, and free-text terms, is provided in Table 2. Search strategies for supplementary sources were adapted according to the syntax and functionality of each platform. Google Scholar was searched using combinations of the main exposure and outcome terms, and the first relevant result pages were screened. Reference lists of included studies and relevant reviews were also manually screened to identify additional eligible articles. The search date of 10 January 2026 refers to the final literature search update performed prior to manuscript resubmission. The 2025 studies were identified during this final update and were screened using the same predefined eligibility criteria and study selection process as all earlier records; no separate post hoc selection process was applied.

### 2.6. Screening and Selection Procedure

All records retrieved from the database and supplementary searches were exported and assessed for duplication before title and abstract screening. Duplicate assessment was performed by comparing available bibliographic information, including article title, author names, year of publication, journal/source, and DOI when available. No duplicate records were identified during this process; therefore, all 1304 retrieved records proceeded to title and abstract screening. The 1304 records comprised 353 records from PubMed/MEDLINE, 879 from Google Scholar, 27 from Wiley Online Library, and 45 from the Clinical Oncology journal collection. Two reviewers independently screened titles and abstracts against the predefined eligibility criteria. Articles deemed potentially eligible were subsequently assessed in full text to confirm inclusion. Disagreements at any stage were resolved through discussion and consensus. The study selection process is summarized in a PRISMA flow diagram (Figure 1).

### 2.7. Data Extraction

Data was extracted from each included study using a standardized extraction sheet. Two reviewers independently extracted the following variables: author, year of publication, study design, total sample size, number of prostate cancer cases, participant age, metabolic syndrome definition and measurement, obesity class, tumor grade and/or stage, progression or recurrence outcomes, inflammatory markers, hormonal data, effect measure (e.g., odds ratio, hazard ratio, relative risk), direction of association, intervention used (when applicable), metabolic syndrome treatment used (when applicable), adjustment models/covariates, and risk of bias information. Any discrepancies between reviewers were resolved through discussion and agreement, with re-review of the original article when needed.

### 2.8. Outcomes and Effect Measures

The primary outcome of this review was prostate cancer risk/incidence in relation to metabolic syndrome. Secondary outcomes included clinicopathologic characteristics (tumor grade and/or stage, including advanced or metastatic disease where reported), disease progression, biochemical recurrence, and survival outcomes. Effect measures extracted from included studies included odds ratios (ORs), hazard ratios (HRs), relative risks (RRs), incidence rate ratios (IRRs), correlation coefficients (r), and median overall survival (mOS), along with corresponding 95% confidence intervals (CIs) or reported measures of variability where applicable. When multiple estimates were presented, the most fully adjusted model was preferentially extracted to minimize confounding, and the direction of association (positive, inverse, or null) was recorded as reported by each study.

### 2.9. Risk of Bias and Quality Assessment Tools

Risk of bias was assessed independently by two reviewers using tools selected according to study design. Cohort studies were assessed using the Newcastle–Ottawa Scale (NOS) for cohort studies, evaluating participant selection, comparability of cohorts, and outcome assessment. Case–control studies were assessed using the NOS for case–control studies, focusing on case definition/selection, control selection, comparability, and exposure ascertainment. Randomized controlled trials were assessed using the Cochrane Risk of Bias 2 (RoB 2) tool, covering bias arising from randomization, deviations from intended interventions, missing outcome data, outcome measurement, and selective reporting. Cross-sectional studies were assessed using the Joanna Briggs Institute (JBI) critical appraisal checklist for analytical cross-sectional studies. Observational clinical studies and multicenter observational studies were assessed using the NOS tool aligned to their structure: studies with longitudinal follow-up were evaluated using the NOS cohort version, while multicenter biopsy-based or clinic-based observational studies without follow-up were evaluated using the JBI analytical cross-sectional checklist. Any discrepancies were resolved through discussion and consensus.

### 2.10. Data Synthesis

Given the heterogeneity across included studies in terms of metabolic syndrome definitions, study populations, outcome measures, and reported effect estimates, findings were synthesized using a narrative (qualitative) approach rather than a pooled meta-analysis. Studies were grouped and summarized by outcome domain, including prostate cancer risk/incidence, tumor aggressiveness and clinicopathologic features (grade and/or stage, including advanced/metastatic disease), and disease outcomes (progression, biochemical recurrence, survival, and mortality). Within each domain, results were compared according to study design, geographic region, metabolic syndrome definition, and degree of confounder adjustment, and the direction of association (positive, inverse, or null) was reported for each study.

To address conceptual heterogeneity, studies evaluating metabolic syndrome as a formal composite exposure were interpreted separately from studies evaluating individual metabolic syndrome components, insulin-resistance indices, inflammatory biomarkers, or hormonal/metabolic profiles. Component-based and biomarker-based studies were retained because they met the predefined eligibility criteria by addressing metabolic dysfunction in relation to prostate cancer outcomes; however, they were not interpreted as equivalent to studies using a formal metabolic syndrome diagnosis. Instead, these studies were used to contextualize possible drivers of the observed association and to help explain heterogeneity across the evidence base.

To provide a more structured qualitative synthesis, extracted findings were further organized according to study design, geographic/population context, metabolic syndrome definition, outcome domain, direction of association, adjustment level, and risk-of-bias appraisal. Where effect estimates were reported, the most fully adjusted estimate was prioritized. Studies that did not report adjusted effect estimates or confidence intervals were summarized descriptively and interpreted with caution. This structured approach was used to identify whether findings were consistent across prespecified evidence categories rather than relying on an unstructured narrative summary.

Forest plots without pooling were considered; however, they were not generated because the included studies reported non-comparable effect measures across different outcomes, exposure definitions, and adjustment models. Instead, structured tabular in the Section 3 were used to summarize direction of association, adjustment level, and interpretive weight across study categories.

### 2.11. Assessment of Heterogeneity

Heterogeneity was assessed qualitatively by examining differences across studies in metabolic syndrome definitions and measurement (e.g., diagnostic criteria used and component thresholds), study design (cohort, case–control, cross-sectional, randomized trial, and multicenter observational designs), population characteristics (age distribution, race/ethnicity, baseline metabolic risk, and screening context), and prostate cancer outcomes and definitions (incidence, grade/stage classifications, recurrence/progression endpoints, and survival measures). Additional sources of heterogeneity were evaluated based on exposure timing (baseline MetS vs. MetS assessed near diagnosis), follow-up duration, and covariate adjustment sets (including differences in adjustment for PSA screening, BMI/obesity, diabetes, and other comorbidities). These factors were considered when interpreting variability in the magnitude and direction of reported associations across studies.

To further structure the assessment of heterogeneity, findings were summarized across three prespecified interpretive domains: metabolic syndrome definition or exposure type, geographic/population context, and prostate cancer detection context. MetS definitions were grouped as formal composite definitions (e.g., NCEP ATP III/ATP III, AHA/NHLBI, WHO, Chinese Diabetes Society, or study-defined ≥3-component criteria), component-count or individual-component approaches, and biomarker/index-based metabolic measures. Population context was considered by broad geographic region and by race- or ethnicity-stratified cohorts where reported. Detection context was categorized according to whether studies were population-based, biopsy-based, surgical/pathology-based, metastatic/clinical cohorts, or biomarker-focused cross-sectional analyses. These subgroup patterns were summarized descriptively in Section 3 rather than statistically because of non-comparable exposure definitions, outcomes, and adjustment models.

### 2.12. Sensitivity Analysis

Sensitivity analyses were planned to evaluate the robustness of the findings by assessing whether conclusions changed when restricting the synthesis to studies using formal or standardized metabolic syndrome definitions, including NCEP ATP III/ATP III, AHA/NHLBI, WHO, Chinese Diabetes Society, or clearly stated study-defined criteria requiring multiple MetS components. Studies using isolated metabolic variables, insulin-resistance indices alone, inflammatory biomarkers, hormonal markers, or molecular/metabolic prognostic indices were considered non-standard or proxy metabolic exposures and were evaluated separately as supportive evidence rather than as direct evidence of formal MetS. Additional sensitivity considerations included restricting interpretation to studies with adjusted effect estimates and giving greater interpretive weight to larger prospective or population-based cohorts. Because the included studies were heterogeneous in outcome definitions and effect measures, sensitivity analyses were conducted qualitatively rather than through pooled quantitative re-analysis.

### 2.13. Publication Bias

Formal assessment of publication bias (e.g., funnel plot asymmetry or statistical tests) was not performed because a quantitative meta-analysis was not conducted and because the included studies were heterogeneous in design, exposures, and outcome measures. Instead, the potential for publication bias was considered qualitatively by noting differences in study size, direction of reported associations, and completeness of reporting across the included literature, and this limitation was acknowledged in the interpretation of findings.

### 2.14. Certainty of Evidence

The overall certainty of evidence was not formally graded using a framework such as GRADE because the included studies were predominantly observational and exhibited substantial heterogeneity in metabolic syndrome definitions, study populations, outcome measures, and adjustment approaches, which limited the ability to generate pooled estimates and apply structured certainty ratings. Therefore, conclusions were based on qualitative synthesis, with emphasis on study design, consistency of findings, precision of effect estimates, and risk of bias when interpreting the strength of the evidence.

## 3. Results

### 3.1. Study Selection

The search identified 1304 records in total, including 353 from PubMed/MEDLINE, 879 from Google Scholar, 27 from Wiley Online Library, and 45 from the Clinical Oncology journal collection. All retrieved records were assessed for duplication by comparing available bibliographic information, including title, author names, year of publication, journal/source, and DOI when available. No duplicate records were identified; therefore, all 1304 records proceeded to title and abstract screening. During title and abstract screening, 1058 records were excluded because they were not relevant to metabolic syndrome and prostate cancer outcomes, were not original studies, or clearly did not meet eligibility criteria. The remaining 246 reports were sought for retrieval, and all were retrieved and assessed in full text. Of these, 222 were excluded because they did not assess metabolic syndrome and/or prostate cancer outcomes (*n* = 195) or were not original studies (*n* = 27). Ultimately, 24 studies met the inclusion criteria and were included in the qualitative synthesis (Figure 1).

### 3.2. Study Characteristics

The 24 included studies comprised a mix of observational and interventional designs, including prospective cohort studies, retrospective cohort studies, observational cohorts, case–control studies, cross-sectional studies, multicenter observational studies, and one randomized controlled trial protocol.

Study sizes ranged from small clinic-based cohorts (e.g., 100–500 participants) to very large population-based cohorts, including a nationwide cohort exceeding 5 million participants and a large prospective cohort with >240,000 participants. Study populations spanned Europe, North America, and Asia, with ages generally in the mid-60s to early 70s in most clinical cohorts and broader adult ranges in population datasets.

Definitions of metabolic syndrome varied across studies but generally relied on a ≥3 of 5 components framework. Components generally included central obesity (waist circumference or BMI criteria), hypertension, dyslipidemia (triglycerides and/or HDL cholesterol), and impaired fasting glucose/diabetes. Some studies focused on insulin resistance indices rather than a binary MetS diagnosis. Outcomes reported included prostate cancer incidence/risk, biopsy detection and clinically significant disease, pathologic aggressiveness (grade/stage), biochemical recurrence, progression-free survival, overall survival/median survival, and in selected studies, inflammatory markers and hormonal profiles. The key characteristics of the 24 included studies are summarized in Table 3.

The main findings of the included studies are summarized by outcome domain in Table 4, including the direction of association and the principal effect estimate reported for each study. This table is intended to provide a concise overview of results across heterogeneous study designs and endpoints.

To further clarify how heterogeneity influenced interpretation, findings were also summarized by MetS definition or exposure type, geographic/population context, and prostate cancer detection setting (Table 5A).

When findings were organized by exposure type and outcome domain, composite MetS showed inconsistent associations with overall prostate cancer incidence, whereas studies focused on clinically significant, high-grade, or advanced disease showed a more consistent positive direction. Recurrence and survival findings were more variable, with null findings in some post-radical prostatectomy cohorts but stronger adverse prognostic signals in metastatic or molecular-index-based studies. Component-level and biomarker-based studies generally supported the interpretation that selected metabolic abnormalities, particularly adiposity, insulin resistance, inflammation, and endocrine-metabolic alterations, may explain part of the observed heterogeneity.

### 3.3. Risk of Bias Assessment Tools

Risks of bias and methodological quality were evaluated to support interpretation of the included evidence and to account for differences in study design and reporting. Because this review included multiple study types, we used design-specific assessment tools: the Newcastle–Ottawa Scale (NOS) was applied to cohort and case–control studies, the Joanna Briggs Institute (JBI) critical appraisal checklist was applied to cross-sectional and observational clinical studies, and the Cochrane Risk of Bias tool (RoB 2) was applied to randomized controlled trials. All assessments were performed independently by two reviewers, and disagreements were resolved through discussion and consensus.

Overall, the risk-of-bias assessment supported inclusion of the studies in the qualitative synthesis, but the ratings should not be interpreted as indicating absence of bias. Although many cohort and case–control studies were rated as good quality overall, several had limitations in comparability, follow-up adequacy, or adjustment for important confounders, including PSA screening history, family history, physical activity, medication use, and other cardiometabolic comorbidities (Table 6 and Table 7). In addition, clinic-based, biopsy-based, and surgical cohorts were particularly vulnerable to selection and detection bias because inclusion often depended on referral, biopsy indication, PSA testing, or treatment pathway. Therefore, associations with clinically significant or aggressive prostate cancer in these cohorts may have been influenced by differential detection rather than reflecting a purely biological relationship. Cross-sectional and biomarker-focused studies were also limited by inability to establish temporality and by variable control for confounding factors. The risk-of-bias assessments are summarized by study design in Table 6, Table 7, Table 8 and Table 9, including cohort studies, case–control studies, the randomized controlled trial protocol, and cross-sectional or observational clinical studies. Accordingly, findings from large prospective cohorts and better-adjusted studies were given greater interpretive weight, whereas results from smaller, retrospective, cross-sectional, or clinic-selected studies were interpreted as supportive or hypothesis-generating rather than definitive. 

When risk-of-bias findings were considered by outcome domain, the interpretive strength of the evidence varied. For incidence/risk outcomes, most studies were rated as good quality using NOS-based assessment, including several large population-based or cohort studies; however, biopsy-based incidence studies remained vulnerable to referral and detection bias. For aggressiveness and clinicopathologic outcomes, the evidence was less robust because several studies were biopsy-based, surgical, cross-sectional, or rated as fair quality, limiting causal interpretation and increasing the possibility that associations with high-grade or clinically significant disease were influenced by PSA testing, biopsy indication, or treatment selection. Prognosis and survival studies included several NOS-rated good-quality cohorts, but many were retrospective or conducted in advanced/metastatic settings, where confounding by indication, baseline disease severity, comorbidity burden, treatment selection, and reverse causality may affect observed associations. Biomarker and component-level studies were largely cross-sectional or exploratory and were therefore interpreted as hypothesis-generating rather than definitive evidence.

### 3.4. Association Between Metabolic Syndrome and Prostate Cancer Outcomes

Because the included studies varied substantially in design, exposure definition, population, and outcome type, the results were interpreted by evidence category rather than as a single uniform association. Studies using a formal metabolic syndrome definition were considered the primary evidence for the association between metabolic syndrome and prostate cancer outcomes. Studies evaluating isolated metabolic variables, insulin-resistance indices, inflammatory markers, or hormonal measures were interpreted as supportive component-level or mechanistic evidence. Overall, the evidence did not show a consistent association between metabolic syndrome as a composite diagnosis and prostate cancer incidence. Signals were more frequently reported for clinically significant, high-grade, advanced, or poorer-prognosis disease than for overall incidence, but these findings were interpreted cautiously because many came from biopsy-based, surgical, retrospective, or metastatic cohorts.

#### 3.4.1. Prostate Cancer Incidence/Risk

In a large prospective cohort from UK Biobank, MetS as a composite exposure was not associated with prostate cancer risk, while individual components showed divergent directions: hypertension (IRR 1.22; 95% CI 1.03–1.44) and obesity (IRR 1.24; 95% CI 1.05–1.46) were positively associated with incidence, whereas prediabetes/diabetes (IRR 0.80; 95% CI 0.67–0.94) and low HDL-C (IRR 0.82; 95% CI 0.69–0.97) were inversely associated; hyperlipidaemia was not associated (IRR 1.07; 95% CI 0.93–1.24) [12]. Given its prospective design, large sample size, and component-level analysis, the UK Biobank study was given substantial interpretive weight in this review. Its null finding for composite MetS, together with divergent associations for individual components, suggests that the binary MetS construct may be too broad to capture the heterogeneous metabolic pathways relevant to prostate cancer incidence [12].

Findings varied across settings and populations. In a multicenter repeat biopsy cohort, a MetS diagnosis was associated with higher odds of prostate cancer detection on repeat biopsy (OR 2.79; 95% CI 1.49–5.22) [14]. In a case–control analysis stratified by race, MetS was marginally associated with increased prostate cancer risk among African American men (OR 1.71; 95% CI 0.97–3.01) but not among Caucasian men (OR 1.02; 95% CI 0.64–1.62), highlighting heterogeneity by population context [26]. This pattern suggests that a binary composite MetS definition may obscure divergent component-level associations, particularly when obesity, hypertension, glycemic status, and lipid parameters influence prostate cancer detection or biology in different directions.

#### 3.4.2. Clinicopathologic Features (Grade/Stage; Advanced/Metastatic)

Several biopsy- and surgery-based studies reported positive associations between MetS and aggressive disease features, although these findings should be interpreted cautiously because the evidence comes from selected clinical cohorts that may be affected by PSA testing patterns, biopsy referral, treatment selection, and detection bias. In a biopsy-based case–control study, Cicione et al. reported that MetS was associated with high-grade prostate cancer (ISUP PGG ≥ 3) (multivariable OR 1.50; 95% CI 1.10–2.56), while no significant association was observed with overall prostate cancer diagnosis [28]. Similarly, in a cross-sectional biopsy cohort, Gómez-Gómez et al. reported that MetS was associated with clinically significant prostate cancer (GS ≥ 7) (adjusted OR 1.83; 95% CI 1.05–3.20), and systemic inflammation was also associated with clinically significant disease (CRP > 2.5 mg/L: OR 2.00; 95% CI 1.14–3.51) [29].

In a radical prostatectomy cohort, Zhang et al. reported associations between MetS and adverse pathology, including GS ≥ 8 (OR 1.67; 95% CI 1.10–2.55), pT3-4 disease (OR 1.58; 95% CI 1.1–2.27), and lymph node involvement (OR 1.75; 95% CI 1.04–2.96) [35]. Guerrios-Rivera et al. also reported an association between MetS components and high-grade prostate cancer in a multiracial biopsy cohort (OR 1.73; 95% CI 1.21–2.48), while associations with overall prostate cancer and low-grade disease were not significant [13]. Overall, these findings suggest a more frequent positive signal for clinically significant, high-grade, or adverse pathological features than for overall diagnosis; however, interpretation remains limited by observational design, selected biopsy/surgical settings, variable adjustment, and potential detection bias.

#### 3.4.3. Prognosis (Progression/Recurrence; Survival/Mortality)

For post-treatment recurrence outcomes, results were inconsistent. In a retrospective cohort, MetS was not significantly associated with biochemical recurrence after radical prostatectomy (HR 0.38; 95% CI 0.13–1.10; *p* = 0.074) [34]. These recurrence-related findings should not be interpreted as directly conflicting because the studies evaluated related but distinct constructs. Xu et al. assessed clinical metabolic syndrome in a post-radical prostatectomy cohort, whereas Ren et al. evaluated a metabolic syndrome-related prognostic index derived from tumor/transcriptomic data. Therefore, the recurrence evidence was interpreted as heterogeneous, with findings depending on whether metabolic dysfunction was measured as a clinical syndrome, a component burden, or a prognostic molecular/metabolic signature [33,34].

A related but distinct approach using a MetS-related prognostic index reported the index as an independent predictor of biochemical recurrence-free survival, with multivariable Cox HR 1.01 (*p* = 0.002) in TCGA and HR 1.75 (*p* = 0.040) in a validation cohort [33].

In advanced disease, some retrospective and real-world studies reported poorer survival metrics among patients with MetS or greater metabolic burden; however, these findings should be interpreted cautiously because prognosis in metastatic cohorts may be influenced by treatment selection, baseline disease severity, comorbidities, and reverse causality. In metastatic prostate cancer, patients with MetS had shorter median progression-free survival (18 vs. 21 months) and shorter median overall survival (38 vs. 62 months) than those without MetS [36]. Another observational clinical study reported shorter outcomes with increasing MetS component burden, with mOS reported as 58 months (≥4 components), 31 months (≤2 components), and 25 months (=3 components) [21].

Finally, broader prognosis signals were supported by a Catalan cohort analysis of life expectancy after cancer diagnosis, where remaining life expectancy at age 68 years was 13.23 years for those with 0 metabolic syndrome components versus 8.92 years for those with ≥3 components (with corresponding life years lost reported) [16].

#### 3.4.4. Components and Biomarker Findings

Several studies evaluated metabolic dysfunction through individual MetS components, insulin-resistance indices, inflammatory biomarkers, or hormonal/metabolic profiles rather than a formal MetS diagnosis. These studies were interpreted as supportive and hypothesis-generating rather than as direct equivalents of composite MetS studies. Overall, component-level findings suggested that selected metabolic abnormalities, particularly adiposity, hypertension, insulin resistance, systemic inflammation, and endocrine-metabolic alterations, may help explain heterogeneity across the evidence base; however, the limited number of studies and differences in measurement approaches preclude firm conclusions [12,18,23,29,32,36].

Insulin-resistance-focused findings, including those reported by Wang et al., suggest that derived metabolic indices may capture risk-related phenotypes not reflected by binary MetS definitions, but these results require confirmation in prospective cohorts with standardized adjustment [18]. Similarly, inflammatory biomarker findings should be considered exploratory, as they were reported in selected populations and were not consistently evaluated across studies [23,29]. Hormonal and metabolic profiling studies may provide mechanistic context, particularly in newly diagnosed or advanced disease settings, but they should not be interpreted as establishing causal pathways between MetS and prostate cancer outcomes [32,36].

#### 3.4.5. Sensitivity Analysis by MetS Definition

When the synthesis was restricted to studies using formal or standardized composite MetS definitions, the overall interpretation remained similar but became more cautious. Evidence for a uniform association between composite MetS and overall prostate cancer incidence remained inconsistent, particularly in light of the large UK Biobank cohort showing no significant association for composite MetS [12]. In contrast, several studies using formal MetS definitions continued to suggest associations with clinically significant, high-grade, or adverse pathological features [28,29,35]. However, recurrence and survival findings remained mixed and were influenced by disease setting, study design, and adjustment level [16,21,33,34,36].

Studies using non-standard metabolic proxies, including insulin-resistance indices, biomarker-based measures, hormonal profiles, or metabolic-syndrome-related prognostic indices, were therefore interpreted separately as supportive and hypothesis-generating evidence [18,23,32,33]. Excluding these proxy-based studies did not materially change the main conclusion that the evidence is heterogeneous and that any association appears more consistent for aggressive disease phenotypes than for overall incidence. However, exclusion of these studies weakened support for mechanistic claims related to insulin resistance, inflammation, and endocrine-metabolic pathways, reinforcing the need to interpret these findings cautiously.

## 4. Discussion

### 4.1. Principal Findings

This systematic review synthesized evidence from 24 studies examining metabolic syndrome and related metabolic abnormalities in relation to prostate cancer risk, clinicopathologic features, and outcomes. The overall evidence was heterogeneous and should not be interpreted as showing a single uniform association between metabolic syndrome and prostate cancer. A central finding is that the large prospective UK Biobank study reported no significant association between composite MetS and prostate cancer incidence, while individual components showed divergent associations [12]. This finding challenges the utility of treating MetS as a binary construct and supports a more component-driven interpretation. Studies using formal composite MetS definitions provided mixed evidence for overall prostate cancer incidence, whereas stronger signals were reported in some studies evaluating clinically significant, high-grade, advanced, or poorer-prognosis disease. However, these findings were interpreted cautiously because many came from retrospective, biopsy-based, surgical, or metastatic cohorts with greater vulnerability to selection bias, detection bias, residual confounding, confounding by indication, and reverse causality.

The added value of the present review should be interpreted as an update and refinement of prior evidence rather than as a complete change in conclusions. Compared with earlier syntheses, including the 2017 meta-analysis by Gacci et al. [37], this review incorporates more recent evidence, including large contemporary cohorts, 2025 publications, component-level metabolic analyses, insulin-resistance indices, biomarker studies, and prognosis-oriented endpoints [12,16,18]. However, because the included studies varied substantially in MetS definitions, populations, outcome definitions, effect measures, and adjustment models, a new meta-analysis restricted to standardized outcomes was not feasible. Thus, the contribution of this review is not to provide a new pooled effect estimate, but to clarify which findings are confirmatory, where newer evidence adds nuance, and why interpretation differs across incidence, aggressiveness, recurrence, survival, and component-level outcomes.

The qualitative sensitivity analysis restricting interpretation to studies using formal or standardized MetS definitions did not materially change the overall conclusion, but it reduced the strength of inference for insulin-resistance, biomarker, and molecular-index findings, which were therefore treated as hypothesis-generating rather than definitive evidence.

### 4.2. Interpretation by Outcome Domain

Across the included evidence, associations between MetS and incident prostate cancer were inconsistent. In the UK Biobank prospective cohort, MetS as a composite exposure was not associated with prostate cancer risk (IRR 1.07; 95% CI 0.94–1.22) [12]. In the same cohort, individual components showed divergent directions (e.g., hypertension IRR 1.22 [1.03–1.44]; obesity IRR 1.24 [1.05–1.46]; prediabetes/diabetes IRR 0.80 [0.67–0.94]; low HDL-C IRR 0.82 [0.69–0.97]; hyperlipidemia IRR 1.07 [0.93–1.24]) [12]. Race-stratified case–control evidence also suggested population-level heterogeneity, where Beebe-Dimmer et al. reported a marginal association between MetS and prostate cancer among African American men (OR 1.71; 95% CI 0.97–3.01) but not among Caucasian men (OR 1.02; 95% CI 0.64–1.62), with stage-stratified estimates also provided [26]. The potential for detection bias may also help explain why incidence associations are less consistent than associations with aggressive disease. This issue is illustrated by the Prostate Cancer Prevention Trial placebo-group analysis reported by Thompson et al., which demonstrated that a substantial proportion of men with PSA levels of 4.0 ng/mL or lower and normal digital rectal examination findings nonetheless harbored prostate cancer, including high-grade disease. Although metabolic syndrome status was not specifically reported in that cohort, these findings are relevant because obesity, a major component of metabolic syndrome, has been associated with lower measured PSA levels through hemodilution, raising the possibility that metabolic dysfunction may obscure PSA-based detection while clinically significant disease remains present [38].

Compared with incidence, several included studies suggested stronger associations between MetS and aggressive or clinically significant disease. In the study by Cicione et al., MetS independently predicted high-grade prostate cancer (multivariable OR 1.50; 95% CI 1.10–2.56; *p* = 0.023) [28]. In the study by Gómez-Gómez et al., MetS was associated with clinically significant prostate cancer (OR 1.83; 95% CI 1.05–3.20), and CRP was also associated with clinically significant disease (CRP > 2.5 mg/L: OR 2.00; 95% CI 1.14–3.51) [29]. Surgical pathology findings were consistent with this “aggressiveness” signal in Zhang et al., where MetS was associated with adverse features including GS ≥ 8 (OR 1.670; 95% CI 1.096–2.545), pT3-4 disease (OR 1.583; 95% CI 1.106–2.266), and lymph node involvement (OR 1.751; 95% CI 1.038–2.955) [35].

For prognosis, findings were mixed and appeared to vary by endpoint and setting. In the study by Xu et al., MetS was not significantly associated with biochemical recurrence after radical prostatectomy (HR 0.38; 95% CI 0.13–1.10; *p* = 0.074) [34]. In contrast, Ren et al. reported that a metabolic syndrome-related prognostic index (MSRPI) predicted biochemical recurrence-free survival, with HR 1.013 (*p* = 0.002) in TCGA and HR 1.752 (*p* = 0.040) in a validation cohort [33]. In metastatic disease, Zhuo et al. reported shorter survival metrics among men with MetS, including median PFS 18 vs. 21 months and median OS 38 vs. 62 months compared with non-MetS patients [36].

Overall, the prognosis evidence should be interpreted as suggestive rather than causal. Findings were mixed across recurrence and survival endpoints, and several prognosis-related studies were retrospective or conducted in metastatic populations, where confounding by indication, treatment selection, baseline disease burden, comorbidity, and reverse causality may influence the observed associations.

The recurrence literature illustrates the importance of distinguishing between exposure definitions. Xu et al. did not identify a significant association between clinically defined MetS and biochemical recurrence after radical prostatectomy, whereas Ren et al. reported prognostic value for a metabolic syndrome-related index. These findings should be interpreted as heterogeneous rather than directly contradictory because the studies measured different constructs: one assessed clinical MetS status, while the other evaluated a derived prognostic index reflecting metabolic-syndrome-related tumor biology. Accordingly, the current evidence is insufficient to conclude that clinical MetS independently predicts biochemical recurrence, although metabolic signatures may still have prognostic relevance [33,34].

Importantly, observed associations between MetS and aggressive or advanced disease should not be interpreted as necessarily causal. Biopsy- and surgery-based cohorts may overrepresent men with abnormal PSA testing, clinical suspicion, or treatment-eligible disease, while differences in biopsy referral, healthcare access, comorbidity burden, and treatment pathways may influence which patients are diagnosed, treated, and followed. These factors may amplify apparent associations between metabolic dysfunction and adverse pathology.

Taken together, the included literature suggests that positive associations are reported more often for aggressive, clinically significant, or advanced disease outcomes than for overall incidence. However, this pattern should be interpreted as suggestive rather than definitive because it is based largely on observational studies with heterogeneous designs, selected clinical populations, variable adjustment, and potential detection bias.

### 4.3. Component-Driven Effects

An important interpretive issue in this review is the distinction between metabolic syndrome as a formal composite diagnosis and related metabolic abnormalities assessed individually or through derived indices. Formal MetS definitions combine multiple cardiometabolic abnormalities into a binary exposure, whereas component-level studies and insulin-resistance indices may capture more specific biological processes. Therefore, component-based and biomarker-based findings should not be viewed as interchangeable with formal MetS studies. Instead, they help identify which aspects of metabolic dysfunction may be most relevant to prostate cancer risk, aggressiveness, or prognosis [12,18,23,29,32,36].

Across the included studies, the direction and strength of the MetS-prostate cancer association often depended on which metabolic component was driving the exposure, rather than the MetS label acting as a single uniform risk state. In the UK Biobank cohort, this was evident because different MetS components showed opposing directions of association within the same dataset, supporting the concept that composite MetS definitions can mask component-specific effects.

Obesity may behave differently across populations. A race-stratified case–control study reported that when individual features were examined, obesity was inversely related to prostate cancer among Caucasians (OR 0.51; 95% CI 0.33–0.80) but not among African Americans (OR 1.15; 95% CI 0.70–1.89), indicating effect modification by population context and/or detection patterns rather than a consistent obesity effect across groups [26].

Insulin resistance appears sensitive to how it is measured. In a cross-sectional study using non-insulin-based insulin resistance indices, the highest vs. lowest quintile showed markedly increased odds of prostate cancer across multiple indices (ZJU index OR 15.592; TyG OR 7.306; TG/HDL-c OR 4.790; METS-IR OR 9.844, all with reported 95% CIs) [18]. This supports the interpretation that insulin resistance burden captured by indices may show stronger associations than binary glycemic labels in some datasets.

Component burden and endocrine dynamics may matter in advanced diseases. An observational clinical study reported outcome differences when stratified by the number of MetS components (mS reported as 58 months for ≥4 components, 31 months for ≤2, and 25 months for 3 components), suggesting cumulative metabolic burden may be clinically meaningful [21]. In metastatic prostate cancer, MetS was also linked to treatment-related hormonal kinetics: MetS was an independent risk factor for time to testosterone nadir > 6 months (OR 2.16; 95% CI 1.08–4.33; *p* = 0.03) [36].

Overall, these component-level findings support discussing MetS as a heterogeneous exposure, where adiposity and insulin-resistance phenotypes, and in advanced settings, the accumulation of multiple metabolic abnormalities may drive observed associations more than the composite diagnosis alone.

### 4.4. Biological Plausibility

Several biological pathways have been proposed to explain potential links between cardiometabolic dysregulation and prostate cancer, including insulin-IGF signaling, systemic inflammation/adipokines, and androgen-steroid hormone pathways. However, the evidence synthesized in this review is largely observational, and many biomarker-focused studies were cross-sectional or conducted in selected clinical populations. Therefore, these mechanisms should be interpreted as plausible explanatory hypotheses rather than as evidence that MetS directly causes prostate cancer development, aggressiveness, or poorer outcomes. Observed associations may also reflect detection bias, differences in PSA screening or healthcare access, residual confounding, treatment selection, or reverse causation.

Insulin resistance and hyperinsulinemia may be biologically relevant through downstream growth-factor pathways, including IGF-related signaling, but the included studies do not establish this pathway as a causal mediator of prostate cancer outcomes. Studies evaluating insulin-resistance indices or metabolic-endocrine variables provide supportive context, but these findings require confirmation in prospective cohorts with standardized exposure assessment, temporality, and adjustment for screening-related and lifestyle confounders [12,18,32].

Similarly, chronic low-grade inflammation and adipose signaling may represent plausible links between metabolic dysfunction and prostate cancer behavior. Some included studies evaluated systemic inflammatory markers, adipokines, or related biomarkers in relation to clinically significant disease or recurrence-related outcomes [23,29]. However, these findings were reported in selected biopsy-based or post-treatment cohorts and should be interpreted as exploratory rather than definitive evidence of inflammatory mediation.

Metabolic dysfunction may also coincide with altered androgen biology and steroidogenesis, which are relevant to prostate cancer biology and treatment response. Studies assessing steroid hormone profiles, testosterone kinetics, or metabolic-syndrome-related prognostic signatures provide mechanistic context, particularly in newly diagnosed or advanced disease settings [32,33,36]. Nevertheless, because these analyses were observational and often conducted near or after diagnosis, they cannot distinguish whether metabolic alterations contributed to tumor behavior, reflected disease burden, or were influenced by treatment and comorbidity.

Beyond the MetS-focused evidence synthesized in this review, emerging literature on prostate cancer biology further supports the need to interpret metabolic risk within a broader molecular and diagnostic context. Studies addressing cancer stem-cell biology, chronic inflammation, molecular driver alterations, urinary biomarkers, docetaxel-related biomarkers, and adiponectin/MetS component-based prediction models provide useful background for precision risk stratification and treatment research [39,40,41,42,43,44]. However, these studies were included only as contextual literature and were not treated as direct evidence in the systematic synthesis unless they met the predefined eligibility criteria for evaluating MetS or related metabolic dysfunction in relation to prostate cancer outcomes.

### 4.5. Limitations

This review has several limitations that should be considered when interpreting the findings. First, the included literature was methodologically heterogeneous, with variability in study design (prospective and retrospective cohorts, case–control studies, cross-sectional analyses, and observational clinical studies), populations, and clinical settings, which limited direct comparability across studies and precludes robust pooling of estimates. Second, definitions and measurements of metabolic syndrome differed across studies (including variations in component thresholds and whether waist circumference, BMI-based criteria, medication use, or laboratory measurements were used), increasing the risk of exposure misclassification and contributing to inconsistent associations across cohorts. Third, several studies were conducted in clinic-based or biopsy/surgical cohorts, which may introduce selection bias and detection bias related to PSA screening practices, referral patterns, and biopsy sampling limitations; these factors can influence both prostate cancer detection and the apparent relationship with metabolic traits. Fourth, although many studies reported multivariable adjustment, residual confounding remains likely, particularly for lifestyle factors (diet, physical activity), medication use (e.g., statins, antihypertensives, glucose-lowering therapies), and screening behavior, which were not consistently measured or adjusted across all studies. Fifth, the presence of multiple cross-sectional and retrospective designs limits causal inference and raises the possibility of reverse causality, especially for biomarker and hormonal analyses measured near or after diagnosis. Finally, publication bias could not be formally assessed because the evidence base was synthesized qualitatively without meta-analysis and included diverse effect measures and endpoints; therefore, the possibility that studies with null findings were less likely to be published cannot be excluded.

Risk-of-bias assessment further indicated that although most studies were suitable for qualitative synthesis, differences in comparability, confounder adjustment, follow-up adequacy, and study design limited the strength of causal inference (Table 6, Table 7, Table 8 and Table 9). Therefore, findings from prospective, larger, and better-adjusted studies were considered more informative than results from smaller, cross-sectional, or clinic-selected cohorts.

Risk-of-bias findings also help explain part of the observed heterogeneity. In particular, associations between MetS and aggressive or clinically significant prostate cancer were frequently reported in biopsy-based or surgical cohorts, where PSA screening patterns, obesity-related PSA hemodilution, biopsy referral, and selection of treatment-eligible patients may influence both detection and apparent disease severity. Similarly, prognosis-related associations in metastatic or retrospective cohorts may reflect treatment selection, baseline disease burden, comorbidities, or reverse causation rather than a direct effect of MetS on survival. Therefore, even when individual studies received an overall favorable quality rating, the direction and strength of associations were interpreted in light of study design, detection context, adjustment quality, and temporality.

Accordingly, biologically plausible mechanisms should be interpreted as explanatory hypotheses rather than evidence that MetS directly causes prostate cancer development, aggressiveness, or poorer outcomes.

Another important limitation is that the search was centered primarily on PubMed/MEDLINE, with supplementary searches of Google Scholar, Wiley Online Library, the Clinical Oncology journal collection, and reference lists. Embase, Scopus, Web of Science, and the Cochrane Library were not searched, which may have reduced search sensitivity and increased the possibility that relevant studies were missed. This limitation is particularly relevant because metabolic syndrome, prostate cancer epidemiology, and biomarker-related studies may be indexed differently across databases. Although supplementary searches and reference-list screening were performed to partially mitigate this limitation, the search strategy should not be interpreted as equivalent to a fully comprehensive multi-database systematic review. Future updates should incorporate broader database coverage and database-specific search strategies across additional bibliographic sources.

Generalizability was also limited by differences in geographic region, population structure, MetS definition, and prostate cancer detection context. Findings from biopsy-based and surgical cohorts may not generalize to population-based screening settings, while findings from European or Asian cohorts may not apply directly to other populations because of differences in body-composition thresholds, metabolic risk profiles, PSA screening intensity, healthcare access, and treatment pathways.

Importantly, an overall “good” rating on the NOS did not eliminate concerns regarding residual confounding, retrospective ascertainment, or selection and detection bias, particularly in clinic-, biopsy-, and surgery-based cohorts.

Because several recently published 2025 studies were identified near the final search update and some used non-standard metabolic proxies rather than formal MetS definitions, the sensitivity analysis by exposure definition was particularly important; however, it remained qualitative because of heterogeneity in outcomes, effect measures, and adjustment models.

### 4.6. Future Directions

Future research should prioritize well-designed prospective cohorts with repeated measurements of metabolic exposures to clarify the temporal relationship between MetS, its individual components, and prostate cancer development and progression. Standardizing MetS definitions and component thresholds across studies (e.g., consistent use of NCEP ATP III or IDF criteria and clear reporting of medication-based definitions) would improve comparability and reduce exposure misclassification.

Given the heterogeneity observed at the component level, studies should move beyond a binary MetS label and use component-specific and severity-based models, including adiposity measures that better capture central obesity (waist circumference/waist–hip ratio) and metabolic markers reflecting insulin resistance rather than diabetes status alone. Future observational work should also explicitly address detection and screening bias by incorporating PSA screening history, prostate volume, and diagnostic pathway variables into adjustment strategies, and by evaluating whether obesity-related PSA dilution or differential health-care utilization influences observed associations.

Mechanistic and translational studies are needed to connect metabolic phenotypes to prostate tumor biology. Integrating inflammatory markers, adipokines, and endocrine profiles with clinical outcomes may clarify pathways linking metabolic dysregulation to aggressive disease features and treatment response. In advanced disease, additional work should assess whether MetS influences androgen-deprivation therapy dynamics, treatment toxicity, and survival outcomes, and whether metabolic interventions modify these trajectories.

Finally, interventional studies targeting metabolic health (dietary patterns, structured physical activity, weight loss strategies, and cardiometabolic risk optimization) should evaluate prostate cancer-relevant endpoints, including progression markers, recurrence, and patient-centered outcomes, to determine whether improving metabolic status translates into clinically meaningful benefits for men at risk for or living with prostate cancer.

## 5. Conclusions

This systematic review of 24 studies suggests that the relationship between metabolic syndrome, related metabolic abnormalities, and prostate cancer is heterogeneous and varies according to exposure definition, population, study design, and outcome domain. Overall, evidence for a uniform association between composite MetS and prostate cancer incidence was inconsistent. Some studies suggested stronger associations with clinically significant, high-grade, or adverse pathological features; however, these findings should be interpreted cautiously because the evidence was largely observational and influenced by heterogeneity in study design, MetS definitions, detection context, confounder adjustment, and risk of bias. Findings for recurrence and survival outcomes were mixed and appeared sensitive to disease setting and endpoint definitions. Collectively, the included literature supports a cautious, component-driven interpretation, particularly involving adiposity- and insulin resistance-related phenotypes, but component-level and biomarker findings remain exploratory. Further standardized prospective studies with careful control for screening-related bias, detection bias, treatment-related confounding, and consistent metabolic definitions are needed to clarify whether metabolic health independently influences prostate cancer risk and outcomes.

## Figures and Tables

**Table 1:** PICO-TT Framework.

Component	Inclusion	Exclusion
Population	For prostate cancer incidence outcomes: Adult men (≥18 years) drawn from the general population or clinical cohorts without restriction to prostate cancer status at baseline. For clinicopathologic features and prognosis outcomes: Adult men (≥18 years) with prostate cancer drawn from clinical, biopsy, surgical, or population-based cohorts.	Pediatric populations; animals and in vitro studies.
Intervention	Metabolic syndrome defined according to established diagnostic criteria (e.g., NCEP ATP III, IDF, WHO, AHA/NHLBI, or study defined criteria that clearly specify required components/thresholds).	Comparators included men without metabolic syndrome.
Comparison	Men without metabolic syndrome.	
Outcome	Primary outcomes: prostate cancer incidence and risk of prostate cancer diagnosis. Secondary outcomes: prostate cancer aggressiveness (e.g., high-grade disease by Gleason/Grade group where reported), advanced disease and/or metastatic disease at diagnosis, biochemical recurrence, prostate cancer-specific mortality, and overall mortality.	Outcomes other than metabolic syndrome and not associated with prostate cancer.
Timeframe	Cohort studies: minimum follow up ≥1 year for outcomes requiring longitudinal assessment (e.g., incidence, recurrence, mortality). No upper limit on follow-up duration. Case–control studies: follow up requirement not applicable.	
Trial Type	Prospective and retrospective cohort studies, case–control studies, randomized controlled trials, cross-sectional studies, multicenter observational studies.	Systematic reviews, meta-analysis, case series, editorials, books, and grey literature.

**Table 2:** Summary of Search Strategies Used Across Databases.

Database/Source	Search Terms/Strings	Search Approach	Filters	Search Result
Google Scholar	“metabolic syndrome” AND “prostate cancer”; “insulin resistance” AND “prostate cancer”; “obesity” AND “prostate cancer”; “metabolic syndrome” AND “prostate cancer” AND aggressiveness; “metabolic syndrome” AND “prostate cancer” AND recurrence; “metabolic syndrome” AND “prostate cancer” AND survival	Supplementary free-text search	None	879
PubMed (Advanced search)	(“Metabolic Syndrome”[Mesh] OR “metabolic syndrome”[tiab] OR “insulin resistance”[tiab] OR “hyperinsulinemia”[tiab] OR obesity[tiab] OR “central obesity”[tiab] OR hypertension[tiab] OR dyslipidemia[tiab] OR hypertriglyceridemia[tiab] OR “low HDL”[tiab] OR “type 2 diabetes”[tiab] OR “fasting glucose”[tiab]) AND (“Prostatic Neoplasms”[Mesh] OR “prostate cancer”[tiab] OR “prostatic cancer”[tiab] OR “prostate carcinoma”[tiab] OR “prostatic carcinoma”[tiab] OR “prostatic neoplasm”[tiab]) AND (risk[tiab] OR incidence[tiab] OR aggressiveness[tiab] OR “Gleason score”[tiab] OR grade[tiab] OR stage[tiab] OR recurrence[tiab] OR survival[tiab] OR mortality[tiab] OR prognosis[tiab])	MeSH and free-text Boolean search	None	353
Wiley Online Library	“metabolic syndrome” AND “prostate cancer”; “insulin resistance” AND “prostate cancer”; “obesity” AND “prostate cancer”; “central obesity” AND “prostate cancer”; “hypertension” AND “prostate cancer”; “dyslipidemia” AND “prostate cancer”; “type 2 diabetes” AND “prostate cancer”	Supplementary free-text search	None	27
Clinical Oncology journal collection	“metabolic syndrome” AND “prostate cancer”; “insulin resistance” AND “prostate cancer”; “obesity” AND “prostate cancer”; “prostate cancer” AND recurrence; “prostate cancer” AND survival	Supplementary free-text search	None	45

**Table 3:** Characteristics of included studies evaluating metabolic syndrome and prostate cancer outcomes.

Author	Setting/Population	Study Design	Participants (N)	PCa Cases (n)	MetS Definition	Main Outcome Domain(s)
Lee 2025 [12]	UK Biobank men	Prospective cohort	242,349	6467	MetS components (≥3/5): WC ≥ 102 cm; TG ≥ 1.7 mmol/L; HDL ≤ 1.03 mmol/L; BP ≥ 130/85 or on treatment; FBG ≥ 5.6 mmol/L	PCa incidence; component-specific associations
López-Jiménez 2025 [16]	Men with cancer diagnosis (including PCa)	Catalan cohort	183,364	30,879	AHA/NHLBI criteria (≥3/5): obesity (>30 kg/m^2^), hypertension, reduced HDL, elevated TG, and hyperglycemia	Remaining life expectancy; life years lost by MetS component count
Ragle 2025 [17]	mHSPC with bone metastasis	Randomized clinical trial protocol	102	102	MetS noted as risk context; body composition assessed (DXA)	Functional outcomes, BMD, QoL, safety
Wang 2025 [18]	Insulin resistance indices	Cross-sectional	1852	354	Non-insulin-based IR indices (e.g., TyG, TG/HDL, MetS-IR, ZJU index)	PCa odds across IR index quintiles; MPV interaction
Häggström 2018 [19]	Sweden population-based	Cohort	126,482	6036	T2DM status	Favorable vs. aggressive PCa incidence
Albai 2020 [20]	T2DM patients with MetS	Retrospective cohort	1027	4	MetS diagnosis (≥3/5 criteria): fasting glycemia ≥ 100 mg/dL or on treatment, abdominal circumference ≥94 cm, BP ≥ 130/85 mmHg or treatment, TG ≥ 150 mg/dL or treatment, HDL < 40 mg/dL or treatment	Malignancy incidence
An 2022 [21]	Real-world clinical PCa center	Observational clinical study	1303	190	Endocrine-related indicators: TG, HDL, FBG; BP; blood glucose	Progression/survival stratified by number of MetS components
Arab 2016 [22]	Men without suspected PCa	Cross-sectional	481	0	FBS, cholesterol, TG	PSA correlations with metabolic parameters
Asmar 2013 [23]	Post-radical prostatectomy	Observational cohort	1428	107	BMI (≥30 kg/m^2^), DM, hypertension	Biochemical recurrence; inflammatory markers
Bayraktar 2023 [24]	Prospective controlled biopsy study	Prospective controlled study	908	270	ATP III criteria	PCa diagnosis on biopsy
Beebe-Dimmer 2007 [25]	African American men	Case control	498	139	Hypertension, DM, WC	PCa risk; tumor grade (Gleason)
Beebe-Dimmer 2009 [26]	Race-stratified	Case control	881	637	Modified ATP III criteria	PCa risk by race and stage
Caliskan 2019 [27]	PCa pathology cohort	Retrospective cohort	117	60	AHA/NHLBI criteria (≥3/5): obesity (>30 kg/m^2^), hypertension, reduced HDL, elevated TG, and hyperglycemia	Final pathology associations
Cicione 2016 [14]	Multicenter biopsy (HGPIN on initial biopsy)	Multicenter observational study	283	84	WHO clinical criteria	PCa detection on repeat biopsy
Cicione 2022 [28]	Biopsy cohort	Case control	955	395	ATP III criteria	PCa diagnosis and high-grade PCa at biopsy
Gómez-Gómez 2019 [29]	TRUS biopsy cohort	Cross-sectional	524	195	ATP III criteria	Clinically significant PCa (GS ≥7) at biopsy
Guerrios-Rivera 2023 [13]	Multi-race biopsy cohort	Cohort	1051	563	MetS components (≥3/5): dyslipidemia, elevated TG, DM, hypertension, obesity	Aggressive PCa (high vs. low grade)
Hammarsten 2004 [30]	Men with high stage/high-grade PCa	Cross-sectional	150,000	299	MetS components: BMI, hypertension, elevated TG, HDL, LDL, total cholesterol	Metabolic/insulin profile vs. advanced/high-grade PCa
Lee 2022 [31]	Age-stratified	Cohort	5,370,614	36,958	Task Force on Epidemiology and Prevention criteria (≥3/5): obesity (≥90 cm), hypertension (≥130/85 or treatment), hyperglycemia (fasting blood glucose ≥100 mg/dL), hypertriglyceridemia (≥150 mg/dL or treatment), low HDL (<40 mg/dL or treatment)	PCa risk by age group; obesity/MetS effects
Lutz 2022 [32]	Newly diagnosed PCa	Cross-sectional	210	103	Glucose, TG, HDL, LDL, total cholesterol	Metabolic and hormonal differences in PCa vs. controls
Ren 2023 [33]	TCGA-based prognostic cohort	Retrospective cohort	400	400	MetS-related prognostic index (MSRPI)	Biochemical recurrence-free survival prediction
Xu 2020 [34]	Post-radical prostatectomy	Retrospective cohort	214	214	Chinese Diabetes Society Criteria (≥3/4): obesity (≥25 kg/m^2^), hypertension (≥140/90 mmHg or treatment), DM (fasting blood glucose ≥6.1 mmol/L or treatment), dyslipidemia (TG ≥ 1.7 mmol/L and/or HDL < 0.9 mmol/L)	Biochemical recurrence after radical prostatectomy
Zhang 2015 [35]	Post-radical prostatectomy	Retrospective cohort	1016	1016	Chinese Diabetes Society Criteria (≥3/4): obesity (≥25 kg/m^2^), hypertension (≥140/90 mmHg or treatment), DM (fasting blood glucose ≥6.1 mmol/L or treatment), dyslipidemia (TG ≥ 1.7 mmol/L and/or HDL < 0.9 mmol/L)	Advanced/aggressive pathology (GS ≥ 8, pT3-4, LN+)
Zhuo 2024 [36]	Metastatic PCa on endocrine therapy	Retrospective cohort	212	212	MetS components: hypertension, BMI, FBG, TG, HDL	PFS/OS; time to testosterone nadir

Abbreviations: UK, United Kingdom; MetS, Metabolic Syndrome; WC, Waist Circumference; TG, Triglycerides; HDL, High-density Lipoprotein; BP, Blood Pressure; FBG, Fasting Blood Glucose; PCa, Prostate Cancer; AHA/NHLBI, American Heart Association/National Heart, Lung, and Blood Institute; mHSPC, metastatic hormone-sensitive prostate cancer; DXA, Dual-Energy X-ray Absorptiometry; BMD, Bone Mineral Density; QoL, Quality of Life; IR, Insulin Resistance; TyG, Triglyceride-Glucose Index; ZJU, Zhejiang University; MPV, Mean Platelet Volume; T2DM, Type 2 Diabetes Mellitus; DM, Diabetes Mellitus; BMI, Body Mass Index; ATP, Adult Treatment Panel; HGPIN, High-Grade Prostatic Intraepithelial Neoplasia; WHO, World Health Organization; TRUS, Transrectal Ultrasound; GS, Gleason Score; LDL, Low Density Lipoprotein; TCGA, The Cancer Genome Atlas; PFS, Progression-Free Survival; OS, Overall Survival.

**Table 4:** Structured Summary of Main Findings by Exposure Type, Outcome Domain, Adjustment Level, and Direction of Association.

Author	Exposure Type	Outcome Domain	Effect Measure	Adjustment Level	Direction of Association	Notes
Lee 2025 [12]	Composite MetS and individual components	Incidence/Risk	Composite MetS was not associated with PCa incidence: IRR 1.07 (95% CI 0.94–1.22). Individual components showed divergent associations	Adjusted	Null/Mixed	Composite MetS did not predict incidence, but component-level findings suggested heterogeneity within the MetS construct
Beebe-Dimmer 2009 [26]	Modified ATP III MetS	Incidence/Risk	African American men: OR 1.71 (95% CI 0.97–3.01); Caucasian men: OR 1.02 (95% CI 0.64–1.62)	Adjusted	Mixed	Findings suggested possible race-related heterogeneity, with no consistent association across groups
Cicione 2016 [14]	WHO-defined MetS	Incidence/Risk after HGPIN	PCa was detected in 41% of men with MetS; MetS and widespread HGPIN were associated with repeat-biopsy PCa detection: OR 2.79 (95% CI 1.49–5.22)	Adjusted	Increased	MetS was associated with increased PCa detection in a selected repeat-biopsy population
Albai 2020 [20]	Clinical MetS in T2DM cohort	Incidence/Risk	PCa occurred in 0.4% of patients with T2DM and MetS; prostate cancer-specific effect estimate/95% CI not reported in extracted data	Descriptive/not clearly adjusted	Increased	Low event count limits interpretation; finding is supportive but weak
Bayraktar 2023 [24]	ATP III MetS	Incidence/Biopsy Diagnosis	PCa was diagnosed in 35% of men with MetS; among diagnosed cases, 64.4% had Gleason score < 7 and 35.6% had Gleason score ≥ 7. Effect estimate/95% CI not reported in extracted data	Not clearly reported in extracted data	Increased	MetS was associated with biopsy-diagnosed PCa, but association with grade was less clear
Beebe-Dimmer 2007 [25]	MetS features	Incidence/Risk and Grade	MetS: OR 1.9 (95% CI 1.2–3.0); hypertension: OR 2.4 (95% CI 1.5–3.7); waist circumference > 102 cm: OR 1.8 (95% CI 1.2–2.9)	Adjusted	Increased	MetS features, particularly hypertension and central obesity, were associated with PCa risk in African American men
Häggström 2018 [19]	T2DM status/metabolic phenotype	Incidence/Favorable vs. Aggressive PCa	No statistically significant association reported in extracted data	Adjusted	Null	Findings did not support a clear association between T2DM-related metabolic status and PCa risk
Lee 2022 [31]	Obesity and MetS components	Incidence/Age-Stratified Risk	BMI > 30 kg/m^2^ was associated with increased PCa risk in the older age group: OR 1.32 (*p* < 0.0001); 95% CI not reported in extracted data	Adjusted	Increased	Metabolic and obesity-related associations appeared age-specific
Cicione 2022 [28]	ATP III MetS	Aggressiveness/High-grade PCa	MetS was associated with high-grade PCa: OR 1.50 (95% CI 1.10–2.56)	Adjusted	Increased	MetS was more strongly associated with high-grade disease than with overall PCa diagnosis
Gómez-Gómez 2019 [29]	ATP III MetS and CRP	Aggressiveness/Clinically Significant PCa	MetS was associated with clinically significant PCa: OR 1.83 (95% CI 1.05–3.20)	Adjusted	Increased	MetS and systemic inflammation were associated with clinically significant disease
Zhang 2015 [35]	Chinese Diabetes Society MetS	Aggressiveness/Adverse Pathology	GS ≥ 8: OR 1.67 (95% CI 1.10–2.55); pT3-4 disease: OR 1.58 (95% CI 1.10–2.27); lymph node involvement: OR 1.75 (95% CI 1.04–2.96)	Adjusted	Increased	MetS was associated with adverse pathological features after radical prostatectomy
Guerrios-Rivera 2023 [13]	MetS components ≥ 3/5	Aggressiveness/High-grade PCa	High-grade PCa: OR 1.73 (95% CI 1.21–2.48); overall PCa: OR 1.17 (95% CI 0.88–1.57); low-grade PCa: OR 0.87 (95% CI 0.62–1.21)	Adjusted	Increased for high-grade disease	MetS was associated with aggressive PCa, with similar findings across Black and non-Black men
Caliskan 2019 [27]	AHA/NHLBI MetS	Aggressiveness/Final Pathology	Descriptive pathology findings only in extracted data; effect estimate and 95% CI not reported	Descriptive/not clearly adjusted	Increased	Findings suggest a possible relationship between MetS component burden and adverse pathology, but effect estimates were not reported
Hammarsten 2004 [30]	MetS components and insulin profile	Aggressiveness/High-stage and High-grade PCa	Hypertension (*p* = 0.026), hypertriglyceridemia (*p* = 0.019), lower HDL (*p* = 0.005), and higher insulin (*p* = 0.019) were associated with high-stage/high-grade PCa; 95% CIs not reported in extracted data	Not clearly reported in extracted data	Increased	Component-level metabolic abnormalities were associated with advanced/high-grade PCa
Xu 2020 [34]	Chinese Diabetes Society MetS	Biochemical Recurrence After Radical Prostatectomy	MetS was not significantly associated with biochemical recurrence: HR 0.38 (95% CI 0.13–1.10; *p* = 0.074)	Adjusted	Null	Clinical MetS did not independently predict biochemical recurrence in this post-prostatectomy cohort
Ren 2023 [33]	MetS-related prognostic index	Biochemical Recurrence-Free Survival	MSRPI predicted biochemical recurrence-free survival: HR 1.013 (*p* = 0.002) in TCGA and HR 1.752 (*p* = 0.040) in the validation cohort; 95% CIs not reported in extracted data	Adjusted	Increased	A metabolic-syndrome-related molecular index showed prognostic value, but this differs from clinical MetS diagnosis
Zhuo 2024 [36]	MetS components	Prognosis/Metastatic PCa Survival	MetS vs. non-MetS: mPFS 18 vs. 21 months; mOS 38 vs. 62 months. MetS was also associated with delayed testosterone nadir > 6 months: OR 2.157; *p* = 0.031. HR/95% CI for PFS/OS not reported in extracted data	Adjusted/real-world cohort	Increased adverse prognosis	MetS was associated with poorer survival outcomes in metastatic PCa
An 2022 [21]	Number of MetS components	Prognosis/Metastatic PCa Survival	Median survival was shorter in the MetS group than the non-MetS group: 27 vs. 58 months; log-rank *p* = 0.044. By MetS score, median 5-year survival was 58 months for score ≤ 2, 31 months for score = 3, and 25 months for score ≥ 4; overall log-rank *p* = 0.005. In multivariable Cox analysis, MetS score 4–5 was associated with higher mortality risk vs. score 0–2: HR 2.826 (95% CI 1.396–5.724; *p* = 0.004), while MetS score 3 was not statistically significant: HR 1.454 (95% CI 0.774–2.729; *p* = 0.244)	Not clearly reported in extracted data	Increased adverse prognosis	Component burden may be prognostically relevant in metastatic PCa, but interpretation is limited by observational design
Asmar 2013 [23]	Obesity, hypertension, diabetes	Biochemical recurrence after radical prostatectomy	Obesity: aHR 1.37 (95% CI 0.92–2.09); hypertension: aHR 1.51 (95% CI 1.01–2.26); diabetes: aHR 0.73 (95% CI 0.40–1.33)	Adjusted	Mixed	Hypertension showed a positive association with recurrence, while obesity and diabetes findings were less consistent
López-Jiménez 2025 [16]	MetS component count	Prognosis/Remaining Life Expectancy After Cancer Diagnosis	Remaining life expectancy at age 68 years was 13.2 years with 0 MetS components vs. 8.9 years with ≥3 components; effect estimate/95% CI not reported in extracted data	Adjusted	Increased adverse prognosis	Greater MetS component burden was associated with reduced remaining life expectancy after cancer diagnosis
Wang 2025 [18]	Non-insulin-based insulin-resistance indices	Biomarker/PCa Risk	Fully adjusted continuous METS-IR was associated with PCa: OR 1.129 (95% CI 1.110–1.149). Q5 vs. Q1 METS-IR also showed increased odds: OR 9.844 (95% CI 6.862–14.121). Other NI-IR indices showed similar positive associations	Adjusted	Increased	Insulin-resistance indices may capture metabolic risk not reflected by binary MetS definitions alone
Lutz 2022 [32]	Glucose, insulin resistance, steroid profiles	Biomarker/Physiology	PCa patients had higher fasting glucose levels than controls: 5.65 ± 0.05 vs. 5.44 ± 0.05 mmol/L; effect estimate/95% CI not reported in extracted data	Not clearly reported in extracted data	Mixed/supportive	Findings suggest metabolic and hormonal differences in PCa patients but do not establish a direct MetS association
Arab 2016 [22]	FBS, cholesterol, triglycerides, BMI	Biomarker/PSA Correlation	No significant association between PSA and TG, FBS, cholesterol, or BMI; effect estimate/95% CI not reported in extracted data	Not clearly reported in extracted data	Null	Metabolic parameters were not significantly correlated with PSA in this cross-sectional population
Ragle 2025 [17]	MetS as risk context; body composition assessed	Biomarker/Functional Outcomes in mHSPC	No direct PCa risk or survival effect estimate reported; 95% CI not applicable	Not applicable	Not applicable	This study provides contextual evidence related to body composition and advanced PCa but does not directly estimate the MetS-PCa association

Abbreviations: MetS, metabolic syndrome; PCa, prostate cancer; IRR, incidence rate ratio; OR, odds ratio; HR, hazard ratio; aHR, adjusted hazard ratio; CI, confidence interval; HGPIN, high-grade prostatic intraepithelial neoplasia; T2DM, type 2 diabetes mellitus; ATP III, Adult Treatment Panel III; AHA/NHLBI, American Heart Association/National Heart, Lung, and Blood Institute; GS, Gleason score; CRP, C-reactive protein; HDL, high-density lipoprotein; TCGA, The Cancer Genome Atlas; MSRPI, metabolic syndrome-related prognostic index; mPFS, median progression-free survival; mOS, median overall survival; FBS, fasting blood sugar; TG, triglycerides; BMI, body mass index; mHSPC, metastatic hormone-sensitive prostate cancer. Table note: Where multiple estimates were reported, the most fully adjusted estimate was prioritized when available. Effect estimates are presented with 95% confidence intervals where reported by the original studies. For studies that did not report adjusted estimates, confidence intervals, *p*-values, or directly comparable effect measures, findings were summarized descriptively and interpreted with caution.

**Table 5:** (**A**) Subgroup Summary of Findings by MetS Definition, Population Context, and Detection Setting. (**B**) Direction-of-Effect Summary by Outcome Domain and Interpretive Strength.

(**A**)				
**Subgroup Domain**	**Category**		**Overall Pattern**	**Interpretation**
MetS definition/exposure type	Formal composite MetS definitions		Mixed findings for overall PCa incidence; more consistent positive direction for aggressive or clinically significant disease	Composite MetS may be more informative for disease phenotype than for overall incidence, but results vary by definition and cohort setting
MetS definition/exposure type	Component-count or individual-component analyses		Divergent associations across obesity, hypertension, glycemic status, and lipid parameters	Component-level findings suggest that the binary MetS construct may mask opposing or heterogeneous effects
MetS definition/exposure type	Insulin-resistance indices/biomarker-based measures		Generally suggestive of association but based on fewer and less comparable studies	Findings are hypothesis-generating and require confirmation in prospective cohorts
Geographic/population context	North American and race-stratified cohorts		Associations varied by race and study setting	Generalizability may differ by race, screening practices, and healthcare access
Geographic/population context	European population-based cohorts		Large prospective evidence showed null association for composite MetS but divergent component effects	Population-based evidence weakens the interpretation of MetS as a uniform incidence risk factor
Geographic/population context	Asian cohorts		Several studies reported associations with advanced pathology or recurrence/prognosis, but many were retrospective or clinical cohorts	Findings may reflect regional differences in MetS criteria, body composition thresholds, screening, and treatment pathways
Detection/clinical context	Population-based cohorts		Less consistent association with overall incidence	These studies may be less affected by biopsy referral bias but still depend on screening practices
Detection/clinical context	Biopsy-based cohorts		More frequent positive associations with PCa detection or clinically significant disease	Findings may be influenced by PSA testing, biopsy indication, and referral patterns
Detection/clinical context	Surgical/pathology cohorts		Associations more often observed with adverse pathology	Generalizability is limited to treated patients and may reflect selection of surgery-eligible cases
Detection/clinical context	Metastatic or clinical cohorts		Worse prognosis reported in some studies	Interpretation is limited by confounding by indication, treatment selection, disease burden, and reverse causality
(**B**)				
**Outcome Domain**	**Overall Direction of Evidence**	**Main Contributing Study Types**	**Overall Interpretive Strength**	**Key Interpretation**
Overall PCa incidence/risk	Mixed/inconsistent	Population-based cohorts, case–control studies, biopsy cohorts	Moderate but heterogeneous	Composite MetS did not show a consistent association with overall incidence; component-level effects varied by cohort and metabolic factor.
Clinically significant/high-grade disease	More often positive	Biopsy-based, case–control, cross-sectional, and surgical cohorts	Low to moderate	Positive associations were more frequent, but interpretation is limited by detection bias, biopsy referral, and surgical selection.
Adverse pathology/advanced disease	More often positive	Radical prostatectomy and pathology cohorts	Low to moderate	Associations with adverse pathology were reported, but generalizability is limited to selected treated populations.
Biochemical recurrence	Mixed/inconsistent	Post-prostatectomy cohorts and molecular-index studies	Low	Clinical MetS did not consistently predict recurrence; molecular or metabolic indices should be interpreted separately from formal MetS.
Metastatic prognosis/survival	Suggestive adverse association	Retrospective and real-world metastatic cohorts	Low	Worse outcomes were reported in some studies, but confounding by indication, baseline disease burden, treatment selection, and reverse causality limit inference.
Component-level/biomarker findings	Exploratory/hypothesis-generating	Cross-sectional, biomarker, and metabolic-index studies	Low	Insulin-resistance indices, inflammatory markers, and hormonal/metabolic profiles may explain heterogeneity but do not establish causality.

(**A**) This subgroup summary highlights that generalizability differs substantially across evidence categories. Population-based studies are more informative for incidence but may be influenced by screening patterns, whereas biopsy- and surgical cohorts are more informative for clinically significant or pathologic disease but are more vulnerable to detection and selection bias. Geographic differences in MetS definitions, body-composition thresholds, PSA screening practices, and healthcare access may also contribute to the variability in findings. Therefore, results from one setting should not be assumed to apply uniformly across populations or clinical contexts. (**B**) Interpretive categories: Mixed/inconsistent = findings vary across studies or components; more often positive = most available studies in that domain reported an adverse association; exploratory = limited or non-standard evidence requiring confirmation.

**Table 6:** Newcastle–Ottawa Quality Assessment Tool for Cohort Studies.

	Criterion	Lee, 2025 [12]	López-Jiménez, 2025 [16]	Häggström, 2018 [19]	Albai, 2020 [20]	Asmar, 2012 [23]	Caliskan, 2019 [27]	Guerrios-Rivera, 2023 [13]	Lee, 2022 [31]	Ren, 2023 [33]	Xu, 2020 [34]	Zhang, 2015 [35]	Zhuo, 2024 [36]	Bayraktar, 2023 [24]
**A**	**Selection**													
	Representativeness of the exposed cohort	⭐	⭐	⭐	⭐	⭐	⭐	⭐	⭐	⭐	⭐	⭐	⭐	⭐
	Selection of the non-exposed cohort	⭐	⭐	⭐	⭐	⭐	⭐	⭐	⭐	⭐	⭐	⭐	⭐	⭐
	Ascertainment of exposure	⭐	⭐	⭐	⭐	⭐	⭐	⭐	⭐	⭐	⭐	⭐	⭐	⭐
	Demonstration that outcome of interest was not present at start of study	⭐	⭐	⭐	⭐	⭐	⭐	⭐	⭐	⭐	⭐	⭐	⭐	
**B**	**Comparability**													
	Comparability of cohorts on basis of the design or analysis controlled for confounders	⭐⭐	⭐	⭐	⭐	⭐⭐	⭐	⭐	⭐⭐	⭐	⭐⭐	⭐⭐	⭐	⭐⭐
**C**	**Outcome**													
	Assessment of outcome	⭐	⭐	⭐	⭐	⭐	⭐	⭐	⭐	⭐	⭐	⭐	⭐	⭐
	Was follow up long enough for outcomes to occur	⭐	⭐	⭐	⭐	⭐			⭐	⭐	⭐		⭐	⭐
	Adequacy of follow up of cohorts	⭐	⭐	⭐	⭐	⭐			⭐	⭐	⭐		⭐	
**D**	**Overall score**	Good	Good	Good	Good	Good	Fair	Fair	Good	Good	Good	Fair	Good	Good

Table note: ⭐ indicates that the study met the criterion and received one point according to the Newcastle-Ottawa Scale. ⭐⭐ indicates that the study received two points for the comparability domain when appropriate adjustment for key confounders was reported.

**Table 7:** Newcastle–Ottawa Quality Assessment Tool for Case–Control Studies.

	Criterion	Beebe-Dimmer, 2007 [25]	Beebe-Dimmer, 2009 [26]	Cicione, 2022 [28]	Cicione, 2016 [14]
A	**Selection**				
	Is the case definition adequate	⭐	⭐	⭐	⭐
	Representativeness of the case	⭐	⭐	⭐	⭐
	Selection of controls	⭐	⭐	⭐	⭐
	Definition of control	⭐	⭐	⭐	⭐
B	**Comparability**				
	Comparability of case and control	⭐	⭐	⭐⭐	⭐⭐
C	**Exposure**				
	Ascertainment of exposure	⭐	⭐	⭐	⭐
	Same method of ascertainment for case and control	⭐	⭐	⭐	⭐
	Non-response rate	⭐	⭐		
D	**Overall score**	Good	Good	Good	Good

Table note: ⭐ indicates that the study met the criterion and received one point according to the Newcastle-Ottawa Scale. ⭐⭐ indicates that the study received two points for the comparability domain when appropriate adjustment for key confounders was reported.

**Table 8:** Cochrane Risk of Bias 2 for Randomized Controlled Trials.

	Ragle, 2025 [17]
Randomization	✅
Deviation from Intended Intervention	🟡
Missing Data	🟡
Measurement of Outcome	✅
Reporting	🟡
Low Risk of Bias	✅
Some Concerns of Bias	🟡

Table note: ✅ indicates low risk of bias for the corresponding RoB 2 domain. 🟡 indicates some concerns of bias for the corresponding RoB 2 domain.

**Table 9:** Joanna Briggs Institute Critical Appraisal Tool for Cross-Sectional and Observational Clinical Studies.

Criterion	Wang, 2025 [18]	Arab, 2016 [22]	Gómez-Gómez, 2019 [29]	Hammarsten, 2004 [30]	Lutz, 2022 [32]	An, 2022 [21]
Were the criteria for inclusion in the sample clearly defined	Unclear	Yes	Unclear	Unclear	Yes	Unclear
Were the study subjects and the setting described in detail	Unclear	Unclear	Yes	Unclear	Yes	Yes
Was the exposure measured in a valid and reliable way	Yes	Yes	Yes	Yes	Yes	Yes
Were objective, standard criteria used for measurement of the condition	Unclear	Yes	Yes	Yes	Unclear	Yes
Were confounding factors identified	Yes	Unclear	Yes	Unclear	Yes	Unclear
Were strategies to deal with confounding factors stated	Yes	Unclear	Yes	Unclear	Yes	Unclear
Were outcomes measured in a valid and reliable way	Unclear	Yes	Yes	Yes	Yes	Yes
Was appropriate statistical analysis used	Yes	Yes	Yes	Unclear	Yes	Unclear
Overall appraisal	Include	Include	Include	Include	Include	Include

## Data Availability

The original contributions presented in this study are included in the article. Further inquiries can be directed to the corresponding author.

## Supplementary Materials

The following supporting information can be downloaded at: https://www.mdpi.com/article/10.3390/cancers18121955/s1, PRISMA 2020 checklist.
